# Supplementary material for: Barriers and facilitators of primary care management of type II diabetes mellitus in the West African sub-region: A scoping review
Source: PLOS Glob Public Health. 2025 May 5;5(5):e0003733. doi: 10.1371/journal.pgph.0003733 (PMC12052127; doi:10.1371/journal.pgph.0003733)
Supplement: S1 Appendix — (DOCX) [file pgph.0003733.s003.docx]

**Appendix 1: Detailed Search Strategy**

**Database: PubMed**

Search date: December 31, 2023

**Search Block 1: Diabetes Terms**

1. "Diabetes Mellitus"[Mesh] OR
2. "Diabetes Mellitus, Type 2"[Mesh] OR
3. diabet*[Title/Abstract] OR
4. "type 2 diabetes"[Title/Abstract] OR
5. "diabetes mellitus"[Title/Abstract] OR
6. T2DM[Title/Abstract] OR
7. NIDDM[Title/Abstract]

**Search Block 2: Primary Healthcare Terms**

1. "Primary Health Care"[Mesh] OR
2. "Delivery of Health Care"[Mesh] OR
3. "Health Services Accessibility"[Mesh] OR
4. "primary care"[Title/Abstract] OR
5. "primary health care"[Title/Abstract] OR
6. "primary healthcare"[Title/Abstract] OR
7. "health care delivery"[Title/Abstract] OR
8. "healthcare delivery"[Title/Abstract] OR
9. "health services"[Title/Abstract] OR
10. "health care access"[Title/Abstract] OR
11. "healthcare access"[Title/Abstract]

**Search Block 3: West African Countries**

1. "Africa, Western"[Mesh] OR
2. (Benin OR "Burkina Faso" OR "Cape Verde" OR "Côte d'Ivoire" OR "Ivory Coast" OR Gambia OR Ghana OR Guinea OR "Guinea-Bissau" OR Liberia OR Mali OR Mauritania OR Niger OR Nigeria OR Senegal OR "Sierra Leone" OR Togo)[Title/Abstract]

**Search Block 4: Quality/Access Terms**

1. "Quality of Health Care"[Mesh] OR
2. "Health Services Research"[Mesh] OR
3. "Patient Acceptance of Health Care"[Mesh] OR
4. (barrier* OR facilitator* OR challenge* OR access* OR utilization OR utilisation OR quality)[Title/Abstract]

**Combined Search Strategy**

(#1 OR #2 OR #3 OR #4 OR #5 OR #6 OR #7) AND (#8 OR #9 OR #10 OR #11 OR #12 OR #13 OR #14 OR #15 OR #16 OR #17 OR #18) AND (#19 OR #20) AND (#21 OR #22 OR #23 OR #24)

**Limits Applied**

- Publication date: 2000/01/01 to 2023/12/31
- Languages: English, French
