# Supplementary material for: Barriers and facilitators of primary care management of type II diabetes mellitus in the West African sub-region: A scoping review
Source: PLOS Glob Public Health. 2025 May 5;5(5):e0003733. doi: 10.1371/journal.pgph.0003733 (PMC12052127; doi:10.1371/journal.pgph.0003733)
Supplement: Attachment — Submitted filename: Response to Reviewers.docx [file pgph.0003733.s007.docx]

Abhiruchi Galhotra, MBBS, MD
Academic Editor
PLOS Global Public Health

Dear Dr Abhiruchi,

Thank you for considering our manuscript for publication in PLOS Global Public Health, and we appreciate the time and effort of the reviewers whose feedback has been valuable. We have carefully revised the manuscript accordingly, and in the table below include point-by-point responses and actions taken to address each comment.

| Reviewer 1 | Response | | | Page, (Lines) |
| --- | --- | --- | --- | --- |
| *"This should be moved to result section and the reason for exclusion of articles should be clear"* | As suggested, we have moved the statement "Twelve papers were included in this scoping review after screening 4,290 documents" to the results section. We have also added a brief explanation of the reasons for article exclusion to clarify our selection process:  “Articles were excluded if they were not conducted in West African countries, did not focus on primary care for diabetes, or failed to report on factors affecting access, utilisation, or quality of care. We also excluded non-empirical articles. This rigorous exclusion process ensured that only relevant empirical studies addressing our specific research questions were included in the final analysis.” | | | 9, (168 – 172) |
| *"mention the (n=?) each study design included"* | We have addressed this in our response to a similar comment from Reviewer #2. The revised text now clearly states the number of studies for each study design. | | | 9 (173- 176) |
| *"add the references for each study and include the study design of each articles, They study can be arranged according to supply side and demand side issues separately, The table looks mixed of both, and the discussion section also can follow the same."* | We appreciate this insightful suggestion. We have revised Table 2 to include references for each study and their respective study designs. Furthermore, we have reorganized the table to separate supply-side and demand-side issues.  Regarding the organization of supply-side and demand-side issues, we would like to clarify that our current discussion section follows this structure. The section "Health System Factors" (lines 315-377) addresses supply-side issues, covering topics such as service delivery, health workforce, financing, and governance. The "Contextual Factors" section (lines 379-486) primarily deals with demand-side issues, including cultural beliefs, gender dynamics, and individual-level barriers. We believe this existing structure provides a clear and comprehensive analysis of these distinct but interrelated aspects of diabetes care in West Africa. | | | Pages 10 – 21, Table 2 |
| Reviewer 2 | | Response | Page, (Lines) | |
| *"The format needs to be the same in the whole document. Also, please check all the grammatical errors."* | | We appreciate your attention to detail. We have thoroughly reviewed the manuscript for formatting consistency and grammatical errors. The document has been carefully edited to ensure uniformity in formatting and to correct any grammatical issues. |  | |
| *"Introduction section needs to be concise, it is vast and vague."* | | Thank you for this valuable feedback. We have thoroughly revised the introduction section, streamlining the content to provide a more focused and targeted overview of the topic. We have eliminated extraneous information and sharpened our focus on the key issues relevant to diabetes care in West Africa. The revised introduction now provides a more concise and clear context for our study.  In effecting these changes, the following references were modified:  Removed:   1. Tesema AG, Ajisegiri WS, Abimbola S, Balane C, Kengne AP, Shiferaw F, et al. How well are non-communicable disease services being integrated into primary health care in Africa: A review of progress against World Health Organization's African regional targets. PLOS ONE. 2020 Oct 22;15(10):e0240984. 2. Galvanizing Action on Primary Health Care: Analyzing Bottlenecks and Strategies to Strengthen Community Health Systems in West and Central Africa \| Global Health: Science and Practice [Internet]. [cited 2024 Feb 11]. Available from: <https://www.ghspjournal.org/content/9/Supplement_1/S47> 3. Koohpayehzadeh J, Azami-Aghdash S, Derakhshani N, Rezapour A, Alaei Kalajahi R, Sajjadi Khasraghi J, et al. Best Practices in Achieving Universal Health Coverage: A Scoping Review. Med J Islam Repub Iran. 2021 Dec 30;35:191.   Added:  Communicable and non-communicable diseases in Africa in 2021/22 \| WHO \| Regional Office for Africa [Internet]. [cited 2024 Oct 6]. Available from: https://www.afro.who.int/publications/communicable-and-non-communicable-diseases-africa-202122 | 3, (50 – 105) | |
| *"If possible, please give the prevalence."* | | We appreciate you highlighting the importance of including prevalence statistics. We have added the following information to the introduction:  "As of 2019, NCDs accounted for approximately 37% of all mortality in the region, up from 24% in 2000, with diabetes being a major contributor alongside cardiovascular diseases, cancers, and chronic respiratory diseases (1,2)."  This addition clarifies the increasing impact of NCDs, including diabetes, in the context of the ongoing burden of communicable diseases in West Africa. | 3, (52 – 57) | |
| *"This needs to be shifted at the end of the introduction section."* | | As suggested, the research questions have been moved to the end of the introduction section.  This placement provides a smoother transition into the methods section and offers readers an immediate sense of the scope of our review. | 5, (97 – 100) | |
| *"Add children also"* | | As suggested, we have corrected the omission of children in the exclusion criteria. | 6, Table 1, Row 4 | |
| *"Add "n" for both of the following"* *"Please mention "n" for all the study designs."* | | We have modified the section as follows:  "Twelve papers were included in this scoping review after screening 4,290 documents. Most of the included studies (n=7) were from Nigeria, with the remaining studies conducted in Ghana (n=4) and Senegal (n=1). The study designs employed across the various studies included qualitative (n=7), quantitative (n=4) and a mixed-method study (n=1), with study populations predominantly involving healthcare providers and patients." | 9, (168 – 180) | |
| *"Please add references along with the name of authors."* *"Title of the study can be removed from the table. (Title is not relevant here)"* *"Please add references"* | | We have revised Table 2 to reflect these suggestions. References have been added alongside author names, titles have been removed, and necessary changes have been made. | Pages 10 - 21 | |

We thank you again for your valuable feedback, which has helped to strengthen our manuscript, and look forward to your decision.

Sincerely,

Abdul-Basit Abdul-Samed on behalf of all authors
