# Supplementary material for: Barriers and facilitators of primary care management of type II diabetes mellitus in the West African sub-region: A scoping review
Source: PLOS Glob Public Health. 2025 May 5;5(5):e0003733. doi: 10.1371/journal.pgph.0003733 (PMC12052127; doi:10.1371/journal.pgph.0003733)
Supplement: Attachment — Submitted filename: Response_to_Reviewers_auresp_2.docx [file pgph.0003733.s008.docx]

1/16/2025

Ari Probandari, PhD
Academic Editor
PLOS Global Public Health

Dear Dr Ari Probandari,

Thank you for considering our manuscript for publication in PLOS Global Public Health, and we appreciate the time and effort of the reviewers whose feedback has been valuable. We have carefully revised the manuscript accordingly, and in the table below includes point-by-point responses and actions taken to address each comment.

**Reviewer #1 Comments:**

| **Comment** | **Response** | **Page**/**Lines** |
| --- | --- | --- |
| "In result section, kindly confirm the exclusion criteria added is not repeated in methodology section." | We have carefully reviewed both the methodology and results sections to ensure consistency in the presentation of exclusion criteria. The criteria are now clearly stated once in the methodology section (Table 1) and referenced appropriately in the results without repetition. | Pages 5-9 |

**Reviewer 3 comments:**

| **Comment** | **Response** | **Page (Lines)** |
| --- | --- | --- |
| Title: Consider rephrasing thus: “Barriers and Facilitators of Primary Care Management of Type II Diabetes Mellitus in the West African Sub-Region: A Scoping Review”.  Abstract | We thank the reviewer for this suggestion and agree that the proposed title better reflects the core focus of our review questions and findings. We have modified the title accordingly. The new title more precisely captures our systematic analysis of barriers and facilitators in primary care management of diabetes in West Africa, aligning directly with our research questions. | N/A |
| Kindly follow the PRISMA-scl checklist as much as possible. For example, in the method sub-section of the abstract, it is required that you state eligibility criteria for the studies included, sources of evidence, and charting methods. Your conclusion should be based on the review questions and objectives. | We have revised the abstract to strictly follow the PRISMA-ScR checklist. The methods section now clearly states: - Eligibility criteria - Sources of evidence - Charting methods - Search strategy - Study selection process | Pages 2-3 |
| Methodology:  It may be good to organize this section using the Arksey and O’Malley framework as follows:  Stage 1: Identifying the research question: Here define the research question(s) and establish the scope of the review  Stage 2: Identify relevant studies: Here describe the inclusive search strategy and how the studies were selected (apply the inclusion and exclusion criteria)  stage 3: Study selection: describe the screening process e.g., from title to abstract then the full text  stage 4: Charting the data  stage 5: Collating, summarizing, and reporting the results  These headings should guide the description of the methodology to ensure rigor. The authors already stated that they plan to use the Arksey and colleague framework, it will be good to stick with it. I can see that some of this information is already in the manuscript, kindly insert the headings and expand on your description while focusing on the PRISMA-scl checklist for each heading | Thank you, we take this point and have restructured the methodology section using Arksey and O'Malley's framework, with clear headings for each stage: - Stage 1: Identifying research questions - Stage 2: Identifying relevant studies - Stage 3: Study selection - Stage 4: Charting the data - Stage 5: Collating, summarizing, and reporting results | Pages 5-8 |
| Clarify inclusion/exclusion criteria contradictions regarding meta-analyses and case reports | We have revised the inclusion/exclusion criteria to remove any contradictions. Secondary research (including meta-analyses) is now clearly listed under exclusion criteria. We have also clarified that only primary research studies were included. The definition of empirical articles has been explicitly stated. | Page 7, Table 1 |
| Authors should specify the full electronic search strategy for at least one database, including any limits used, such that it could be replicated or repeated. | We have added Appendix 1 containing the complete search strategy for PubMed, including all search terms, Boolean operators, and limits used, ensuring replicability. | Supporting Information (S3) |
| Kindly highlight the research gaps in the topic before charting the path forward in the discussion section | We have added a new section titled "Research Gaps" before "The Path Forward" in the discussion section, systematically outlining: - Geographic limitations in current evidence - Implementation research gaps - Patient perspective gaps - Digital health intervention evidence gaps - Multisectoral approach evaluation needs | Page 36-37 |

| **Comment** | **Response** | **Page (Lines)** |
| --- | --- | --- |
| "The second research question is centered around the mechanism through which these factors work. I am not sure how this was answered in the review." | We appreciate this insightful observation. Thank you. We have strengthened the Results section to make the mechanisms more explicit while avoiding redundancy. Specifically, we have:  1) Revised key sections to clearly articulate how different factors operate through distinct mechanisms: - The financial mechanism showing how lack of insurance creates direct barriers to access (p.31) - Service delivery mechanism demonstrating how inadequate facilities and fragmented care affect outcomes (p.31-32) - Cultural mechanism illustrating how beliefs influence care-seeking pathways (p.33) - Psychological mechanism revealing the bidirectional relationship between distress and care outcomes (p.36-37)  2) Added language to show how these mechanisms interact, creating compound effects on diabetes care  3) Maintained the existing structure while making mechanisms more explicit, allowing the Discussion to flow naturally from these established mechanisms and situate them within broader literature These revisions better answer our second research question by clearly demonstrating how factors work to influence diabetes care in West Africa. | Pages 31-37 |

We thank you again for your valuable feedback, which has helped to strengthen our manuscript, and look forward to your decision.

Sincerely,

Abdul-Basit Abdul-Samed on behalf of all authors
